# A New Tumorsphere Culture Condition Restores Potentials of Self-Renewal and Metastasis of Primary Neuroblastoma in a Mouse Neuroblastoma Model

**DOI:** 10.1371/journal.pone.0086813

**Published:** 2014-01-22

**Authors:** Dongliang Cao, Satoshi Kishida, Peng Huang, Ping Mu, Shoma Tsubota, Masaaki Mizuno, Kenji Kadomatsu

**Affiliations:** 1 Department of Biochemistry, Nagoya University Graduate School of Medicine, Nagoya, Japan; 2 Center for Advanced Medicine and Clinical Research, Nagoya University Hospital. Nagoya, Japan; The University of Hong Kong, Hong Kong

## Abstract

Tumorsphere culture enriches and expands tumor cells, thus providing important resources for cancer studies. However, as compared with metastatic tissues, primary tumors in the nervous system rarely give rise to long-surviving tumorspheres, thereby seriously limiting studies on these cancers. This might be due to the limited self-renewal capability of tumor cells and/or to inappropriate culture conditions. The growth and maintenance of tumor cells may depend on microenvironments and/or cell origins (e.g., primary or metastatic; stem cell-like or progenitor-like). Here, we attempted to establish a tumorsphere culture condition for primary neuroblastoma (NB). Primary tumors in *MYCN* transgenic mice, a NB model, could be serially transplanted, suggesting that these tumors contain cells with a high self-renewal potential. However, primary tumors did not give rise to tumorspheres under a serum-free neurosphere culture condition. The newly established culture condition (named PrimNeuS) contained two critical ingredients: fetal bovine serum and β-mercaptoethanol were essential for tumorsphere formation as well as indefinite passages. The spheres could be passaged more than 20 times without exhaustion under this condition, exhibited a property of differentiation and formed tumors *in vivo*. Unexpectedly, PrimNeuS revealed that the *MYCN* transgenic mice had bone marrow metastasis. Furthermore, subcutaneous tumors derived from tumorspheres of primary tumors showed bone marrow metastasis. Taken together, PrimNeuS provides resources for the study of NB and can be used as a powerful tool for the detection of minimal residual disease and for *in vitro* evaluation prior to personalized therapy.

## Introduction

Tumorsphere culture provides important resources for cancer studies, since it enriches and expands tumor cells. However, the efficiency with which long-surviving tumorspheres are established from primary tumors in the nervous system is not satisfactory, whereas tumorspheres are obtained from metastatic tissues with relative ease. For example, only half of primary high-grade gliomas are able to give rise to tumorsphere lines [1]. Low-grade gliomas rarely give rise to tumorspheres. It is also hard to obtain tumorsphere lines from primary neuroblastomas (NBs), in contrast to those from metastatic tumors [2]. These low efficiencies of tumorsphere formation from primary tumors seriously limit studies on these cancers.

Tumor cells *in vivo* may reside in a microenvironment suitable for maintenance and growth. Such microenvironments may differ between tumor types and origins, e.g., primary or metastatic; stem cell-like or progenitor-like. This may be why a common protocol for tumorsphere cultures cannot be established. Therefore, if appropriate conditions were provided, tumorspheres could be grown *in vitro*. We addressed this hypothesis in the present study by employing *MYCN* transgenic mice, a NB model.

NB is the most common pediatric extracranial solid tumor and is derived from sympathetic neurons [3]. Despite intensive multimodal therapy, high-risk NB patients with relapse in bone marrow have less than a 10% chance of survival [4]. To overcome this problem, it is particularly important to verify the mechanisms of tumor initiation and the metastasis of NB, which remain elusive.

Here, we have established culture condition for long-surviving tumorspheres from primary NBs. This culture condition restores the potential for self-renewal and metastasis of primary NBs.

## Materials and Methods

### Ethics Statement

This study was carried out in strict accordance with the recommendations in the Guide for the Care and Use of Laboratory Animals of the National Institutes of Health. This study was approved by the Animal Care and Use Committee of Nagoya University Graduate School of Medicine (Permit Number: 24371). All efforts were made to minimize suffering.

### Primary Culture of Tumorspheres from Allograft Tumors and Primary Tumors of *MYCN* Transgenic Mice

The procedure was similar to that used in a previous report [5]. Briefly 0.5-cm^3^ of tumor tissue was dissected from an allograft tumor of 25 generations or from a primary tumor of *MYCN* transgenic mice. After washing, the tissue was minced and digested with 0.25% trypsin (Sigma) for 15 minutes, and the digestion was stopped by adding trypsin inhibitor (Sigma). The cells were centrifuged and washed twice. The supernatant was collected into a new tube and centrifuged. To eliminate the red blood cells, the pellet was treated with RBC lysis buffer (Biolegend) according to the manufacturer’s instructions. The cells were then ready for culture. To get tumorspheres from allograft tumors, cells were cultured as previously reported, with DMEM/F12ham (Sigma) plus EGF 10 ng/ml, bFGF 15 ng/ml (Peprotech), 2% B27 supplement, and 1% penicillin/streptomycin (PS, GIBCO), which can be called medium #1. To get tumorspheres from the primary tumors, some other medium cocktails were investigated: medium #2, DMEF/F12ham, 15% FBS (Hyclone), 2% B27 supplement, 1% PS; medium #3, medium #1 plus 15% FBS; medium #4 [6], [7], medium #3 plus 1% non-essential amino acid (NEAA), 1% sodium pyruvate, 55 µM β-mercaptoethanol [8]; medium #5 is medium #4 without FBS; and medium #6 is medium #4 without β-mercaptoethanol. Cells were cultured in a nontreated petri dish in a 37°C, 5% CO_2_ tissue culture incubator. Successfully formed tumorspheres were digested with trypsin and dissociated into single cells by pipetting with a 1 ml tip and were passed every 3–4 days.

### Tumorsphere Growth Assay

The self-renewal ability of tumorspheres was evaluated by clonogenic assay. Cells were dissociated by trypsin treatment and filtered through a 70 µm nylon mesh, then stained with trypan blue. The viable cells were counted with a hemocytometer under a microscope, and 1000 cells were seeded into 24-well nontreated petri dish. Five days later, the spheres were counted. For sphere size, pictures were taken under a phase-contrast microscope. The sphere diameter was measured by Metamorph 6.1 software (Molecular Devices); 100 randomly selected tumorspheres were measured for each group if possible. To perform a proliferation assay, 10^5^ dissociated cells were seed into a 6 cm petri dish. Five days later, the spheres were dissociated into single cells and stained with trypan blue. The viable cells were counted.

To evaluate the enrichment of the stem cells with the new condition, the sphere-forming potential of both the tumor cells of *MYCN* hemizygous transgenic mice and the tumorsphere cells passaged 4 times in the new condition was checked by ELDA (extreme limiting dilution analysis) [9]. Briefly, after dissociation cells were seeded in a 96 well-plate at a density of 640, 320, 160, 80, 40, 20, 10 or 5 cells/well in 200 µl culture medium (6 wells per group). Another 100 µl medium was added 3 days later. At one week, spheres in each well were counted, and the sphere-forming potential was calculated following the instruction of ELDA.

### Induced Differentiation of Tumorspheres and Immunofluorescence Staining

Spheres were collected at low centrifuge speed, about 300–400 rpm, and were washed once to remove dead cell debris. They were then re-suspended with medium containing DMEM/F12ham, 1% FBS, 2% B27 supplement, 1% sodium pyruvate, 10^3^ units/ml LIF, and 1% PS. The spheres were seeded into poly-D-lysine/laminin/fibronectin pre-coated four-well chamber slides (BD). After 12 hours, the medium was changed to differentiation medium: DMEM/F12ham, 1% FBS, 2% B27 supplement, 1% N2 supplement, 50 ng/ml NGF, 50 ng/ml NT3 and 1% PS. The medium was changed everyday. After 3 days, differentiated cells were fixed and checked by immunofluorescence staining.

### *In vivo* Assays of Tumorigenicity and Immunohistochemistry

First, 10^4^ sphere cells were mixed with PBS containing 30% Matrigel (BD Biosciences) and subcutaneously inoculated into 1-month-old 129/SVJ WT mice in both flanks. Three weeks later, the tumors were dissected and weighed. The tumor volume was measured every week using a digital caliper. Volume was calculated as V = height×width2/2. To compare the tumor formation potential, the primary tumor cells, the corresponding primary tumor sphere cells, allograft tumor cells and the corresponding allograft tumor sphere cells were injected subcutaneously into 4 to 5 week-old wild-type mice at different cell numbers, that is 10^4^, 10^3^, 10^2^, 10 cells/200 µl 30% matrigel, and investigated for 1.5 months to evaluate the potential. For histological evaluation, tissues were fixed in 4% paraformaldehyde, dehydrated and embedded in paraffin according to the standard procedure. The samples were cut into 5 µm sections and stained with hematoxylin and eosin for routine checks. After deparaffinage, immunohistochemistry was carried out by blocking endogenous peroxidase activity and then incubating antigen retrieval sections with anti-TH (CHEMICON, AB125), Sox2 (Abcam, ab97957), or Pax6 (Abcam, ab5790) overnight at 4°C following incubation with biotin-conjugated goat anti-rabbit or anti-mouse secondary antibody (BD Pharmingen) for 30 minutes at room temperature. Signals were amplified using the Vectorstain ABC kit (Vector Laboratories) and detected by DAB (Dako) following the manufacturers’ instructions.

### Evaluation of Bone Marrow Metastasis

Tumorsphere cells were dissociated into single cells and transfected with EF-promoter-Venus vector through lentivirus. The virus was packaged as previously reported [5]. After FACS sorting, the cells were passaged twice *in vitro* and then 10^5^ cells were inoculated into syngenic 129/Svj wild type mice. 6 weeks later, the bone marrow cells from both femoral bones were collected, and dissociated into single cell suspension with 25G needles and filtered through 70 µm nylon mesh. The cells were then cultured under the new condition. To examine metastasis of *MYCN* transgenic mice, 3-month-old wild type and hemizygous mice were sacrificed and the bone marrow cells were cultured with a method as described above.

### Total RNA Extraction and RT-PCR

Total RNA was extracted by the Total RNA Extraction Miniprep System Kit (VioGene Biosciences) following the manufacturer’s instructions. After DNaseI treatment, the same amount of RNA was reverse-transcribed into cDNA with the ReverTra Ace qPCR® RT Kit (TOYOBO). Real-time PCR reaction was carried out with the Thunderbird SYBR qPCR Mix kit (TOYOBO) on Stratagene Mx3005P system. The primers used are listed in Table 1.

**Table 1:** Primers used in PCR experiments.

	Forward	Reverse
TH	cagagttggataagtgtcaccac	gggtagcatagaggcccttca
Hand2	tcaacagcgccttcgccgagct	ttgtcgttgctgctcactgtgc
Phox2b	gaccaccagagcagtccgtacg	agtgctgtcgggatcagtgctc
Phox2a	caattcgtacgattcgtgcgtg	acctgcacgcgagcctcagtga
NPY	tggactgaccctcgctctat	gatgagggtggaaacttgga
SYP	ctcctcggctgaattctttg	acagggtccctcagttcctt
Musashi	atggtggaatgcaagaaagc	taggtgtaaccaggggcaag
Sox2	ctctgcacatgaaggagcac	atgtaggtctgcgagctggt
Pax6	agttcttcgcaacctggcta	gaagtcgcatctgagcttcat
C-myc	gcccagtgaggatatctgga	atcgcagatgaagctctggt
Klf4	ccaaagaggggaagaaggtc	agtgcctggtcagttcatcg
Oct3/4	cacgagtggaaagcaactca	agatggtggtctggctgaac
Snail	gaggacagtggcaaaagctc	ggagaatggcttctcaccag
GAPDH	ggaggccatgtaggccatga	ggtggtgaagcaggcatctg

### Statistical Analysis of Data

Statistical significance was evaluated by the two-tailed Student’s t-test. P<0.05 was considered to indicate a significant difference. Results are expressed as means±SD.

## Results

Traditionally, tumorspheres from nervous system tumors have been cultured in serum-free conditions developed to support normal neural stem cells (NSCs) [10]. Normal NSCs expand when cultured under neurosphere culture conditions, but normal progenitors typically do not [11]. Indeed, a serum-free neurosphere culture condition works well to form tumorspheres from metastatic bone marrow tissues of human NBs [2].

**Figure 1:**
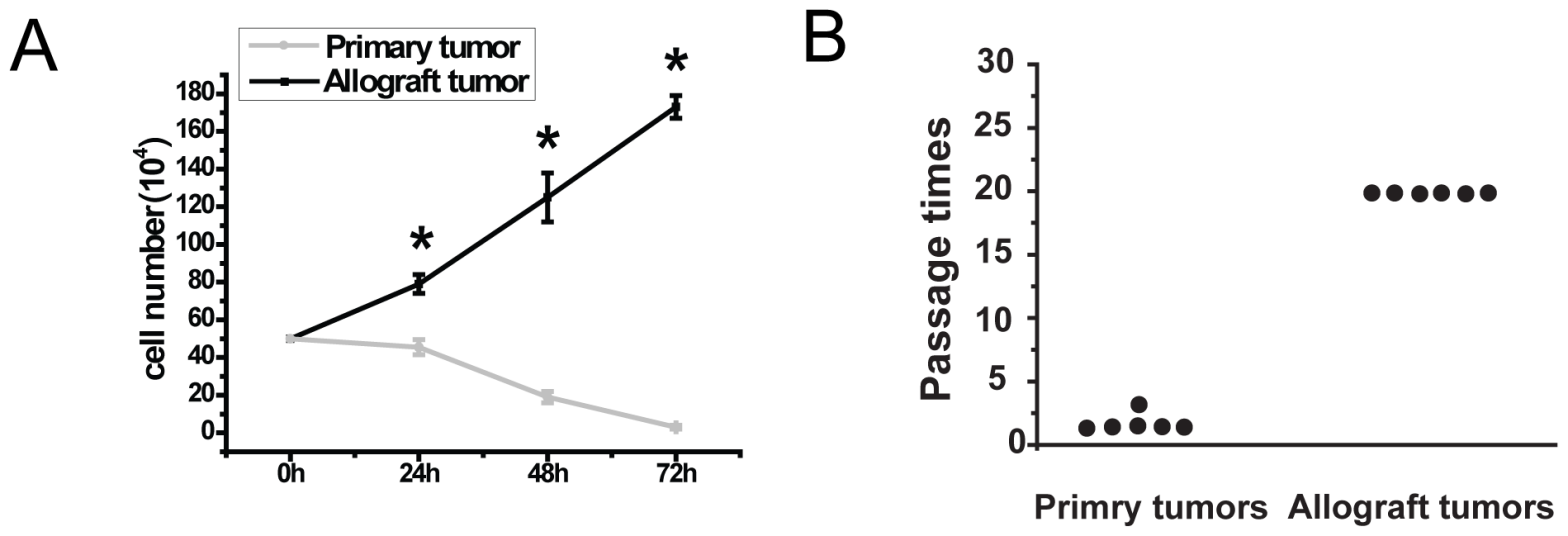
NSC condition can support the long-term survival of tumor cells from allograft tumors but not from primary tumors of MYCN transgenic mice. A, 5×10^5^ cells freshly isolated from allograft tumors and primary tumors were cultured under NSC condition. Cells were counted every day. *P<0.05. B, Both kinds of cells were passaged every 3 days until exhaustion. Passage was stopped after 20 times.

Serial xenotransplantation can increase the population of TICs [12], [13]. We previously established an allograft tumor model by serially allografting minced tumors of *MYCN* Tg mice [5]. Tumorspheres were formed and grew rapidly from allograft tumors, but not primary tumors, of *MYCN* Tg mice under a serum-free neurosphere culture condition (Fig. 1A). Tumorspheres from allograft tumors showed no exhaustion after 20 passages (Fig. 1B). However, this culture condition did not support the growth of tumorspheres from primary NBs (Fig. 1A,B).

**Figure 2:**
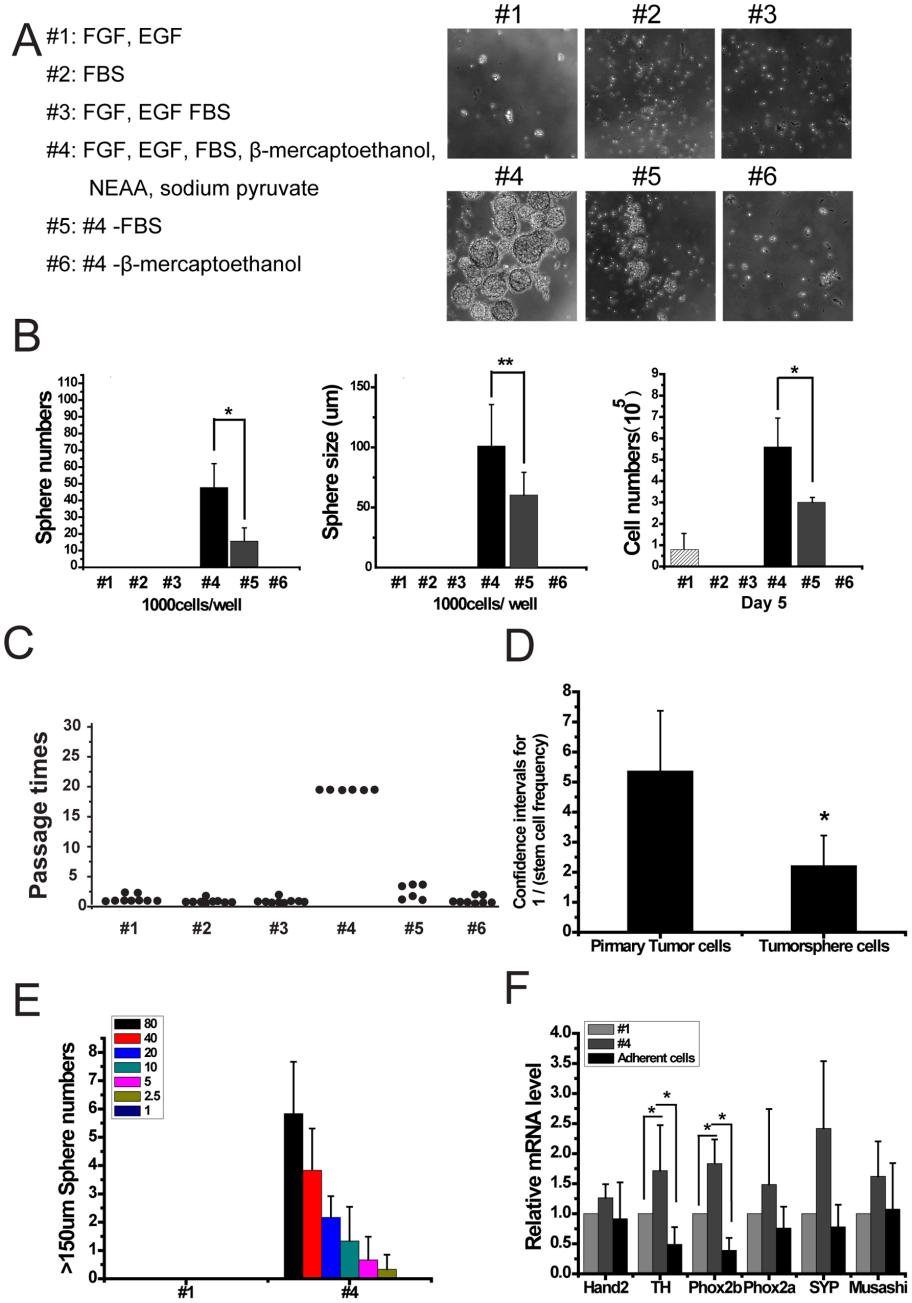
A suitable condition for tumorspheres from primary tumors. A, Important ingredients and a representative photo are shown for each culture condition. Photos were taken at day 5 after culturing in a 24-well plate at 10^3^ cells/well. B, Quantitative results of tumorsphere formation. For proliferation assay, 10^5^ cells were cultured under the indicated conditions in 6-cm petri dishes for 5 days, and the cells were then counted with trypan blue. Representative results of three independent experiments are shown here. *P<0.05, **P<0.001. C, Passageable number of tumorspheres. Culture was stopped after 20 passages. D, Self-renewal capacity of tumor cells and tumorsphere cells after several passages was evaluated by ELDA method described under Materials and Methods. *P<0.05. E, Sphere-forming efficiency of #4 medium. We did a limited dilution adjusting cell density to 1 to 80 per well and incubated the cells for 7 days, and compared sphere (with a diameter more than 150 µm)-forming efficiency under different culture conditions, i.e., #1 and #4. F, Real-time PCR results of sphere cells cultured in #1 and #4 condition and adherent cells. For adherent cell culture, cells were seeded in 10 cm-dish coated with PDL/laminin/fibronectin. Cells were maintained with medium comprising D/F, 2% B27, 1% FBS, β-mercaptoethanol. Medium was changed 24 h later to D/F, 2% B27, 20 ng/ml NGF, 10 ng/ml NT3. mRNA was collected after 3 days culture. *P<0.05.

Next, we sought for a suitable culture condition for tumorspheres from primary NBs. First, we compared four different conditions: #1, serum-free neurosphere medium containing EGF, bFGF and B27; #2, medium containing 15% FBS and B27; #3, #2 plus EGF and bFGF; #4, #3 plus non-essential amino acids (NEAA), sodium pyruvate and β -mercaptoethanol. The #1, #2 and #3 conditions did not support tumorsphere formation from primary NB tissues of *MYCN* Tg mice (Fig. 2A and B, #1-3). In contrast, tumorspheres formed well in the #4 condition (Fig. 2A and B, #4). The #4 medium resembled the medium for pluripotent stem cells, e.g., embryonic stem cells. But leukemia inhibitory factor (LIF) was omitted in this medium, because LIF rather enhanced differentiation and thus inhibited the long-term passage of sphere cells (data not shown).

Next, we asked which ingredients were important in the #4 medium. We found that FBS and β-mercaptoethanol were indispensable for sphere formation and long-term survival. Thus, medium without FBS showed less sphere formation (Fig. 2A and B, #5). This medium did not support indefinite passaging (Fig. 2C, #5). Medium without β-mercaptoethanol did not form spheres (Fig. 2A–C, #6).

We employed ELDA (extreme limiting dilution analysis) [9] to evaluate the sphere-forming potential of both tumor cells freshly prepared from tumors of *MYCN* hemizygous transgenic mice and tumorsphere cells passaged 4 times in #4 medium. We found that about 5 cells freshly isolated from primary tumors could form a sphere, whereas about 2 cells of tumorspheres grown in #4 medium were enough to form a sphere (Fig. 2D). The data suggest that cells with self-renewal capacity were enriched in #4 medium.

Next, we performed a head-to-head comparison of sphere (with a diameter more than 150 µm)-forming efficiency under different culture conditions, i.e., #1 and #4. We did a limited dilution adjusting cell density to 1 to 80 per well and incubated the cells for 7 days. We found that cells cultured in #1 medium did not form spheres, whereas #4 medium supported sphere formation (Fig. 2E). Taking into account that the size of spheres counted was big enough, our data suggest that #4 medium increased the sphere-formation efficiency.

We then compared the properties of sphere cells cultured in #1 and #4 medium and adherent cells. As cells could hardly survive for a long time in #1 medium, we collected all the samples after 3 days culture. As summarized in Fig. 2F, all the NB markers (Hand2, TH, Phox2a, Phox2b and SYP) and the neural crest stem cell marker Musashi tended to increase in sphere cells cultured in #4 medium as compared with sphere cells cultured in #1 medium and adherent cells.

Together, the results allowed us to conclude that the #4 condition supported long-term culture of tumorspheres from primary NB tissues. We call this the culture condition for primary neuroblastoma-derived spheres, or PrimNeuS hereafter.

**Figure 3:**
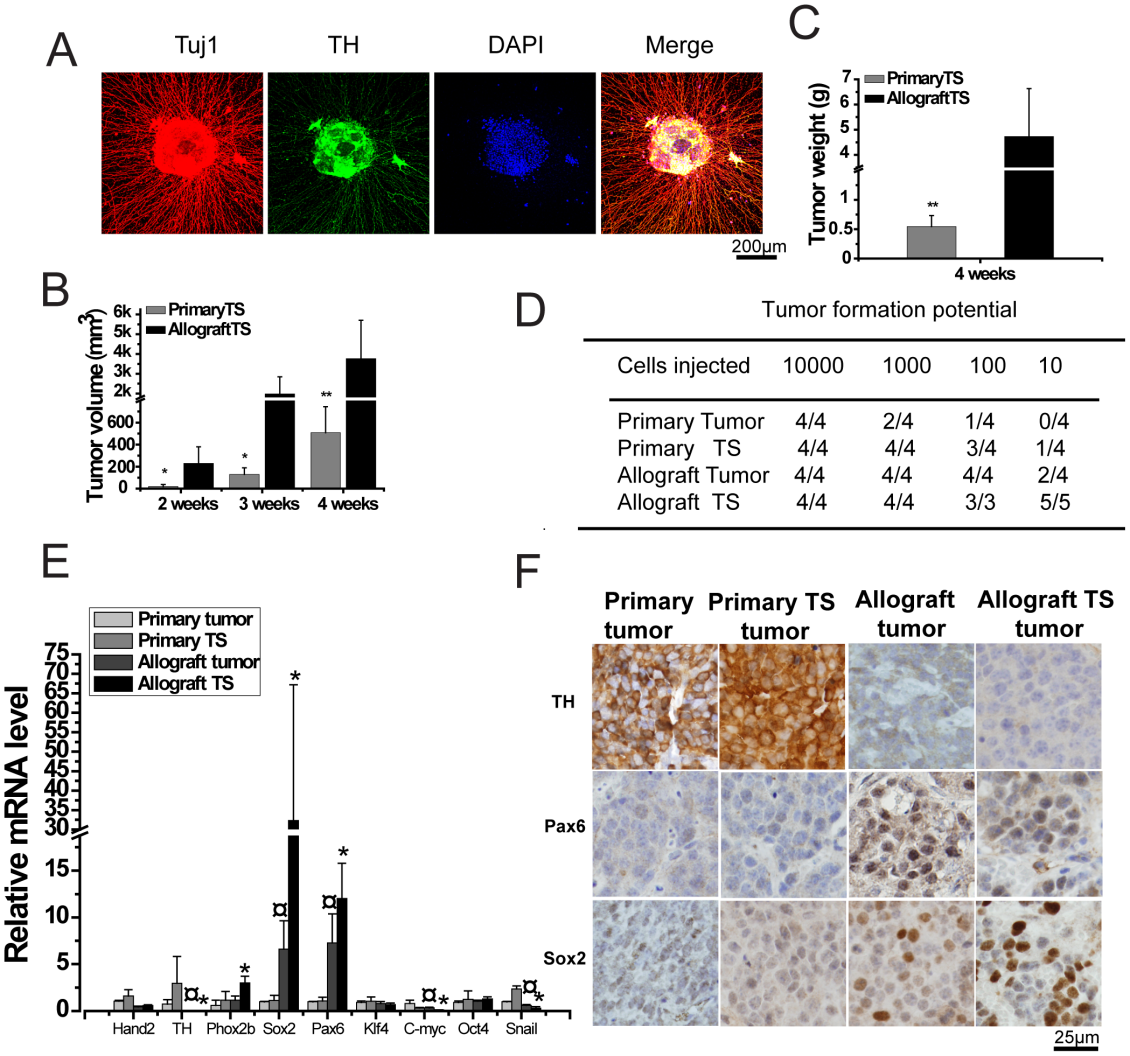
Tumorsphere cells have a differentiation capacity. A, Long radial neurites grew out of tumorspheres under differentiation condition. Representative photos are shown. B, C, Tumorspheres were maintained under PrimNeuS. Totally 10^4^ tumorsphere cells together with 30% matrigel were subcutaneously inoculated into wild-type syngenic mice, tumor volumes and weights were monitored. *P<0.05, **P<0.005. D, The primary tumor cells, the corresponding primary tumor sphere cells, allograft tumor cells and the corresponding allograft tumor sphere cells were injected subcutaneously into 4 to 5 week-old wild-type mice at different cell numbers, and investigated for 1.5 months to evaluate the potential. E, mRNA expression profile of primary tumors, tumorspheres from corresponding primary tumors, allograft tumors and tumorspheres derived from corresponding allograft tumors. The allograft tumorshperes were maintained under the traditional serum-free condition (#1 medium), and the tumorspheres from primary tumors were maintained under PrimNeuS (#4 medium). ¤ P<0.05, primary tumor vs. allograft tumor. *P<0.05, primary TS vs. allograft TS. F, Immunohistochemistry of tumors. Representative photos are shown.

Sphere cells cultured under PrimNeuS had a differentiation property, since radial neurites grew out of these cells under a differentiation condition for 3 days (Fig. 3A). The sphere cells also gave rise to tumors after subcutaneous injection (Fig. 3B, C). We next compared the tumor formation potential of the tumor cells and the corresponding tumorsphere cells from both the primary tumors and allograft tumors. Consistent with Fig. 3C and 3D, allograft tumor cells and their tumorsphere cells showed stronger tumor forming potentials than primary tumor cells and their tumorsphere cells (Fig. 3D). Furthermore, tumorsphere cells from primary tumors needed less cell number to give rise to tumor as compared with tumor cells from primary tumors, supporting the idea that sphere cells are more tumorigenic (Fig. 3D). A set of genes are differentially expressed in primary tumors, tumorspheres from primary tumors, allograft tumors and tumorspheres from allograft tumors (Fig. 3E). Among these genes, it was characteristic that TH, a differentiation marker for sympathetic neurons, was more expressed, and that the stem cell markers Pax6 and Sox2 were less expressed in primary tumors and their spheres (Fig. 3E). These expression profiles were maintained in tumors derived from tumorspheres of allograft or primary tumors (Fig. 3F).

**Figure 4:**
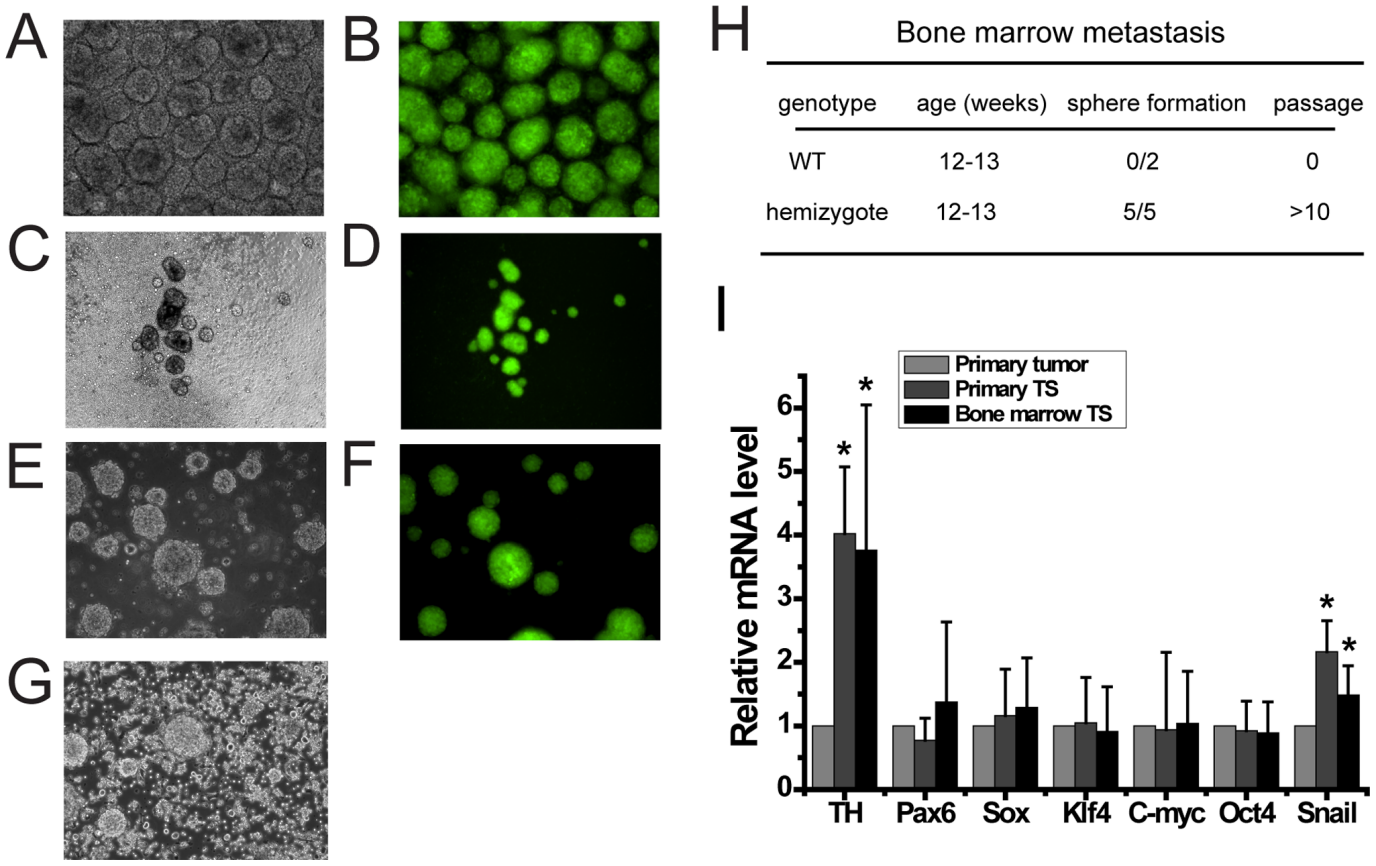
PrimNeuS can support the survival of metastasized cells of neuroblastomas. A–F, Primary tumorspheres were labeled with EF-promoter-Venus by lentivirus, and Venus-positive cells were purified by FACS sorting after 2 passages (A, B). Cells were then subcutaneously inoculated into wild-type mice. One and a half month after inoculation, the bone marrow cells from both femoral bones were cultured under PrimNeuS (C, D). These formed spheres were further expanded under PrimNeuS (E, F). G, The bone marrow of MYCN transgenic mice can form spheres in PrimNeuS medium. H, Bone marrow metastases presented in MYCN transgenic, but not wild-type mice. I, Real-time PCR results of the gene transcripts listed. *P<0.05, primary tumors vs. primary tumorsphere or bone marrow tumorspheres.

We next asked whether or not sphere cells from primary tumors of *MYCN* Tg mice could metastasize. To answer this, we used spheres from primary tumors after three passages and infected them with lentivirus carrying an EF-promoter-Venus expression vector. After two more passages, we sorted Venus-expressing sphere cells followed by another two passages (Fig. 4A, B). Cells were then subcutaneously injected into syngenic 129/SvJ wild-type mice. Bone marrow cells were analyzed 6 weeks later, after subcutaneous tumors were observed. We detected a few clusters of Venus- and TH-positive cells in the femoral bone section by immunohistochemistry (data not shown). Next, we tried to determine the percentage of the positive cells on the bone marrow cells by FACS, but it was below the detectable level. However, in PrimNeuS medium, Venus-positive cells grew and formed spheres (Fig. 4C, D). These spheres could be passaged (Fig. 4E, F). In contrast, PrimNeuS did not support sphere formation of normal bone marrow cells (data not shown). We concluded that sphere cells from primary tumors metastasize to the bone marrow. Consistent with this, we found that PrimNeuS supported sphere formation from the bone marrow of *MYCN* transgenic mice harboring sympathetic ganglia-derived primary tumors, but not from that of wild-type mice (Fig. 4G, H). These spheres could be passaged under PrimNeuS (Fig. 4H). TH and Snail showed significantly higher expression in both tumorspheres from primary tumor (Primary TS) and tumorspheres from bone marrow (Bone marrow TS) as compared with primary tumors, suggesting that primary TS and bone marrow TS have similar properties (Fig. 4I).

## Discussion

Primary tumors in *MYCN* transgenic mice could be serially transplanted without exhaustion (we called these serially transplanted tumors allograft tumors), suggesting that these primary tumors contain cells with a high self-renewal potential. Nevertheless, primary tumors of *MYCN* transgenic mice did not give rise to tumorspheres in a serum-free neurosphere culture condition, which is commonly used for tumorsphere cultures for neural tumors. In contrast, allograft tumors gave rise to tumorspheres in this condition. These phenomena are reminiscent of those of clinical samples; tumorspheres are rarely obtained from primary or low-grade neural tumors, in contrast to metastatic or high-grade tumors [1], [2], [14]. In this study, we established the new culture condition PrimNeuS, by which tumorspheres could be formed from a primary tumor and passaged indefinitely. Thus, this study has shown the self-renewal capacity of primary NB cells *in vitro*.

Barrett et al. recently reported that self-renewal does not predict tumor growth potential in mouse models of high-grade glioma [15]. Thus, while Id1^low^ cells generate tumors more rapidly than Id1^high^ cells, Id1^low^ cells show non-self-renewal capacity in a neurosphere culture condition. However, there is still a possibility that Id1^low^ cells might show self-renewal *in vitro* if an appropriate culture condition were provided. PrimNeuS has enabled us to estimate self-renewal capacity *in vitro*. Furthermore, PrimNeuS revealed for the first time that tumorspheres from primary tumors (Fig. 4A–F) as well as primary tumors themselves (Fig. 4G–I) have the potential for metastasis to the bone marrow. This is unexpected, since bone marrow metastasis has not been reported in the model of *MYCN* transgenic mice. PrimNeuS could be applicable as a sensitive tool to detect tiny bone marrow metastases or minimal residual disease of NB.

Personalized therapy has become a realistic concept for cancer treatment. For example, anti-GD2 antibody-treated NB patients lacking HLA class I ligands for their inhibitory killer cell immunoglobulin-like receptors have significantly higher survival rates than those with HLA class I ligands. Unlicensed NK cells are thought to be responsible for this phenomenon [16]. If tumorspheres are obtained from every patient regardless of tumor status, i.e., primary or metastatic, high risk or low risk and high grade or low grade, those are precious resources for evaluating the efficacy of the treatment prior to its clinical use. Although PrimNeuS medium tended to allow a longer culture of tumorspheres from human NB as compared with the classical serum-free medium (#1 medium) (data not shown), a limited number of samples did not allow us to statistically validate the usefulness of PrimNeuS for human NB. Further evaluation or improvement of the culture condition for human NB will facilitate studies of stem cells of NB.

To successfully eradicate tumors, it may be necessary to eliminate both the TICs and their more differentiated progeny [17], [18]. In glioma, the capacities for self-renewal and tumor initiation are not necessarily restricted to a uniform population of stem like cells, but can be shared by a lineage of self-renewing cell types expressing a range of markers of forebrain lineage [1]. In this context, it was interesting to find in our study that tumorspheres from primary tumors and allograft tumors express distinct markers, but both show tumorigenecity. The success of culturing long-term tumorspheres from primary NB tumors may open new avenues to identify novel stem cell markers for diagnostic and therapeutic NB.

We found that FBS is essential for the formation and indefinite passaging of tumorspheres. In the case of embryonic stem cells, they differentiate into neurons if FBS is removed from the medium. In our case, the decreased concentration of FBS facilitated the outgrowth of neurites of primary tumor spheres, supporting the idea that FBS strongly inhibits neural differentiation of these cells [7], [19], [20]. Our results suggest that FBS helps to keep NB cells undifferentiated. In addition, we found that β-mercaptoethanol was critical for tumorsphere formation. If β-mercaptoethanol was removed after several passages, the tumorspheres were no longer passaged. This suggests that β-mercaptoethanol is also essential for indefinite passaging. The mechanisms underlying β-mercaptoethanol’s functions remain to be verified.

PrimNeus supported the sphere formation from primary tumors and bone marrow in a neuroblastoma model. In contrast, it did not support the sphere formation from normal bone marrow cells. Therefore, PrimNeus may provide an appropriate culture condition for a subset of tumor cells in neuroblastoma (e.g., tumor initiating cells), but may not be generally applicable to stem cells such as normal bone marrow stem cells. This suggests that an appropriate culture condition may depend on cell types. Indeed, hematopoietic stem cells or progenitor cells require several growth factors, such as insulin, IL-3, IL-6, G-CSF and GM-CSF, but these are dispensable for the culture of tumor initiating cells [21], [22], [23].
